# The Value of Serum Immunoglobulin Free Light Chain Assessment in Patients with Monoclonal Gammopathies and Acute Renal Failure

**DOI:** 10.5505/tjh.2012.48640

**Published:** 2012-12-05

**Authors:** Mustafa Cirit, Atilla Üzüm, Pınar Özen, Banu A. Şentürk, Giray Bozkaya, Bahriye Payzin, Orçun Ural

**Affiliations:** 1 İzmir Atatürk Training and Research Hospital, Department of Nephrology, İzmir, Turkey; 2 İzmir Atatürk Training and Research Hospital, Department of Biochemistry, İzmir, Turkey; 3 İzmir Training and Research Hospital, Department of Biochemistry, İzmir, Turkey; 4 İzmir Ataturk Training and Research Hospital, Department of Hematology, İzmir, Turkey; 5 Manisa State Hospital, Department of Internal Medicine, Manisa, Turkey

**Keywords:** Multiple myeloma, Acute renal failure, Immunoglobulin free light chain

## Abstract

**Objective:** Immunoglobulin free light chain (FLC) abnormalities are common in patients with monoclonal gammopathies and the kidneys are the most affected organs. Immunoassays that provide quantitative measurement of FLC in serum indicate monoclonal FLC production based on the presence of an abnormal FLC kappa:lambda (κ:λ) ratio. The aim of this study was to assess the utility of serum FLC measurement as a diagnostic tool for detecting plasma cell dyscrasias in comparison to standard assays, and to ascertain its sensitivity and specificity in patients with acute renal failure (ARF).

**Material and Methods:** Sera from 82 patients with ARF were assessed using serum protein electrophoresis (SPE), serum immunofixation electrophoresis (SIFE), and FLC measurement. The sensitivity and specificity of the FLC ratio in identifying which ARF patients had multiple myeloma (MM) was compared to those of SPE and SIFE.

**Results:** Among the 82 patients with ARF, 7 were diagnosed as MM using SPE, SIFE, and bone marrow biopsy techniques. In total, 8 patients did not have a FLC κ:λ ratio that was within the published reference range (0:26-1:65); the FLC κ:λ ratio based on FLC measurement had a specificity of 96% and sensitivity of 71%, and positive and negative predictive values of 62.9% and 97.3%, respectively, for the diagnosis of MM.

**Conclusion:** The sensitivity and specificity of the FLC κ:λ ratio for diagnosing MM in patients that presented with ARF were lower than those of SPE and SIFE. To further delineate the utility of the FLC κ:λ ratio additional prospective, well-designed large-scale studies are needed.

**Conflict of interest:**None declared.

## Abstract (tr)

**Amaç:** Monoklonal gammopatili hastalarda immunglobulin hafif serbest zincir anormalliklerine sık rastlanır ve bu hastalarda böbrekler en sık etkilenen organlardan biridir. Serum immunglobulin hafif serbest zincir düzeylerinin sayısal ölçümünü sağlayan yöntemlerle, monoklonal immunglobulin hafif serbest zincir üretiminin varlığı anormal κ/λ oranlarına dayanmaktadır. Bu çalışmada akut böbrek yetmezliği ile başvuran hastalarda serum hafif serbest zincir analizinin plazma hücre diskrazilerinin saptanmasında tanısal değerinin standart yöntemlerle karşılaştırılarak, spesifite ve sensitivitesinin değerlendirilmesi amaçlanmıştır.

**Gereç ve Yöntemler:** Akut böbrek yetmezliği ile başvuran 82 hasta serum protein elektroforezi, serum immunfiksasyon elektroforezi ve hafif serbest zincir ölçüm yöntemleriyle değerlendirildi. Akut böbrek yetmezlikli multipl myelom tanılı hastalarda hafif serbest zincir oranının sensitivite ve spesifitesi serum protein elektroforezi, serum immunfiksasyon elektroforez yöntemleriyle kıyaslandı.

**Bulgular:** Akut böbrek yetmezliği ile başvuran 82 hastanın 7’sinde serum protein elektroforezi, immunfiksasyon elektroforezi ve kemik biopsisi ile multipl myelom tanısı kondu. Hastaların 8’inde κ/λ oranı yayınlarda bildirilen referans değerlerinin (0.26-1.65) dışında saptandı; serbest hafif zincir ölçümlerine dayanarak κ/λ oranı myelom tanısı için %96 spesifite,%71 sensitivite, %62.9 pozitif prediktif değer ve%97.3 negatif prediktif değere sahipti.

**Sonuç:** Akut böbrek yetmezlikli multipl myelomlu hastalarda serum protein elektroforezi, serum immunfiksasyon elektroforez yöntemleriyle kıyaslandığında κ/λ hafif serbest zincir oranları düşük sensitivite ve spesifite değerine sahipti. Bu yöntem için daha doğru yorum yapabilmek için daha iyi dizayn edilmiş, prospektif ve çok sayıda hasta içeren çalışmalara ihtiyaç vardır.

## INTRODUCTION

Multiple myeloma (MM) is a malign disease characterized by abnormal proliferation of plasma cells. Monoclonal immunoglobulin secreted by these cells can be quantitatively measured to monitor the disease course [1,2]. The kidneys are the most commonly affected organs in patients with MM. Impaired renal function is present in 22%-43% of MM patients and is associated with survival. The most common pathogenetic lesion that causes renal failure is the so-called myeloma kidney, which is the result of light chain cast nephropathy, and occurs in approximately 65% of patients with myeloma. Among patients diagnosed as myeloma, 25% present with acute renal failure (ARF) [3,4,5,6]. Because renal dysfunction has an impact on survival, early diagnosis is critically important in patients with MM, as it can help prevent renal damage. 

Until recently, serum free light chain (FLC) measurement has been used for the diagnosis and screening of monoclonal gammopathy, light chain amyloidosis (AL), and MM [7,8,9,10]. Determination of monoclonal FLC production is based on the presence of an abnormal FLC kappa:lambda (κ:λ) ratio (reference range: 0:26-1:65) [11]. The aim of the present study was to determine the utility of serum FLC measurement as a diagnostic tool for detecting plasma cell dyscrasias, as compared with standard assays, and to determine its sensitivity and specificity in patients with ARF.

## MATERIALS AND METHODS

**Study design and participants **

The study protocol was approved by the Izmir Ataturk Training Hospital Ethics Committee. The study included 82 patients with ARF that were admitted to hospital between May 2008 and December 2008. Exclusion criteria were age <50 years, renal ultrasonography findings indicative of obstruction and chronic kidney disease, diabetic nephropathy, polycystic kidney disease, pregnancy, malignancy, and collagen tissue diseases. Renal ultrasonography, renal function testing, serum protein electrophoresis (SPE), serum immunofixation electrophoresis (SIFE), and FLC measurement were performed in all the patients. If indicated, bone marrow aspiration and biopsy were performed. We estimated the glomerular filtration rate (GFR) using the Modified Diet in Renal Disease (MDRD) equation. The decision to initiate dialysis was made by a consultant nephrologist based on the following indications: uremia, hyperkalemia, metabolic acidosis, and fluid overload (defined as an estimated GFR [eGFR] <15 mL·min–1·1.73 m–2, according to the MDRD equation). Diagnosis of MM was made by a consultant hematologist in accordance with international diagnostic criteria. ARF was diagnosed based on medical history, physical examination, biochemical parameters, AKIN criteria, and ultrasound findings. Detailed ARF causes cannot be stated because of inconvenience of renal biopsy application. 

**Laboratory assessment**

After a 12-h fasting period, venous blood samples were obtained from the participants and serum was separated via centrifugation at 3000 rpm for 15 min. Samples were stored in small aliquots at –200 °C until analyzed. The serum free light chain concentration was measured using a commercially available latex-enhanced immunoassay kit (Freelite^®^ Human Lambda/Kappa Free Kit, The Binding Site Ltd., Birmingham, UK) and an Olympus AU640 autoanalyzer (Olympus, Tokyo, Japan). The assay consists of 2 separate measurements: one quantifies λκ FLC and to the other quantifies κ FLC. In addition to measuring λ and κ FLC, the assay determines the FLC κ:λ ratio (the reference ranges used [0:26-1:65] were supplied by the manufacturer). Patients with an FLC κ:λ ratio >1:65 have excess κ FLC and are presumed to be producing clonal λ FLC. Patients with a FLC κ:λ ratio <0:26 have excess λ FLC and are presumed to be producing clonal λ FLC. 

SIFE was performed using a Hydrasys Automate, according to the manufacturer’s instructions, and Hydragel 15 and 30 protein(e), and Hydragel 2 and 4 immunofixation gels (Sebia, Lisses, France). 

Serum BUN and creatinine levels were determined using photometric methods, an Olympus AU 640 autoanalyzer (Olympus, Tokyo, Japan), and commercial kits. 

A Toshiba Famia S ultrasound machine was used to assess the urinary system in all patients. Renal parenchymal echogenicity and urinary tractus were assessed. If indicated, bone marrow aspiration and biopsy were performed; samples were fixed with 10% formalin and stained with Hematoxylin-Eosin, and then histopathological evaluation was performed via light microscopy.

## STATISTICAL ANALYSIS

Statistical analysis was performed using SPSS v.15.0. Patient groups were compared using the Mann-Whitney U test, Student’s t test, and chi-square test. Pearson’s correlation test was used for correlation analysis. Statistical significance was set at a P value of less than 0.05.

## RESULTS

Patient baseline demographic and biochemical data are shown in Table 1. Mean age of the patients was 69 ± 9 years; 53.7% were male. Renal ultrasonography findings were normal in all the patients. Among the 82 patients that presented with ARF—confirmed based on medical history, and laboratory and ultrasonographic findings—7 were diagnosed as MM via SPE, SIFE, and bone marrow biopsy findings. Among the 8 patients that did not have an FLC κ:λ ratio within the published reference range (0:26- 1:65), 5 had an FLC κ:λ ratio >1:65 and 3 had an FLC κ:λ ratio <0:26 (Figure). The FLC κ:λ ratio based on FLC measurement had a specificity of 96% and sensitivity of 71%, and positive and negative predictive values of 62.9% and 97.3%, respectively, for diagnosing MM. 

Characteristics of the patients with an abnormal FLC κ:λ ratio are shown in Table 2. Among the 5 patients that had an FLC κ:λ ratio >1:65, 2 were diagnosed as MM, whereas 3 of the patients with an FLC κ:λ ratio <0:26 were diagnosed as MM. Characteristics of patients that had a normal FLC κ:λ ratio and were diagnosed as MM (false negative) are shown in Table 3. In all, 1 of 2 patients with normal SPE and SIFE findings was diagnosed as MM based on bone marrow biopsy findings and had normal FLC measurements indicating the diagnosis of non-secretory myeloma, whereas the other patient had a normal FLC κ:λ ratio and was as diagnosed as MM based on SPE, SIFE, and bone marrow biopsy findings. 

Table 4 shows the comparison between the patients with and without MM. Age, BUN, creatinine, λ, and the FLC κ:λ ratio did not differ between the 2 groups, but the κ value was significantly different. When considering SPE and SIFE findings together, the specificity, sensitivity, and positive and negative predictive values were higher than those for the FLC κ:λ ratio and SPE+FLC κ:λ ratio co-assessment (Table 5). Correlation analysis between age, eGFR, FLC κ, and FLC λ values showed that there was a positive correlation between FLC κ and λ values (P = 0.027, r = 0.87) and a negative correlation between eGFR and FLC κ values (P = 0.027, r = –0.25). The 2 patient groups assessed according to eGFR as 15ml/min after exclusion of MM patients. There wasn’t a difference between FLC λ and the FLC κ:λ ratio, but BUN, creatinine, and FLC κ values significantly differed between the 2 patient groups, with respect to eGFR (Table 6). Pearson’s correlation analysis showed that creatinine was positively correlated with FLC κ (P = 0.019, r = 0.27) and FLC λ (P = 0.018, r = 0.272) levels, and eGFR was inversely correlated with FLC κ (P = 0.023, r = –0.262) and FLC λ (P = 0.034, r = –0.246) levels. Furthermore, the FLC κ level was positively correlated with FLC λ (p<0.001, r = 0.908) and the FLC κ:λ ratio (p<0.001, r = 0.543).

## DISCUSSION

Recently, an assay for serum FLC became commercially available for use as a marker for diagnosing and screening monoclonal gammopathies [7,8,9,10]. There are 3 indications for using serum FLC to diagnose and screen MM and related diseases. First, screening serum using SPE and SIFE with FLC levels is highly sensitive and eliminates the necessity of using 24-h urine collection for the diagnosis of related diseases, except for light chain amyloidosis. Second, FLC assessment has prognostic importance for all plasma cell dyscrasias. Third, FLC assay provides insight into the screening of oligo-secretory plasma cell dyscrasias, quantitatively [9,12,13]. 

Most kidney disease associated with monoclonal gammopathies is caused by monoclonal FLCs, which is a consequence of the kinetics of FLC clearance from the serum by the kidneys. As such, any structural and functional abnormalities in the kidneys and/or excess production of FLCs can lead to deposition and precipitation in situ [8]. The mechanisms involved in the nephrotoxicity of monoclonal FLCs are activation of inflammatory mediators in proximal tubules, proximal tubule necrosis, acquired Fanconi syndrome due to FLC deposition, cast nephropathy, light chain amyloidosis, and light chain deposition disease [14,15]. 

There are limited data concerning the use FLC assays in patients that present with renal disease. In general, as renal clearance decreases, FLC κ and λ increase, but no change occurs in the FLC κ:λ ratio; however, different reference ranges must be evaluated in patients with renal disease in order to increase the specificity and sensitivity. Some patients with plasma cell dyscrasias may present with renal pathologies, and delayed identification and treatment leads to irreversible renal failure [8,10]. The present study aimed to evaluate the sensitivity and specificity of the FLC κ:λ ratio for detecting paraproteinemias in patients that presented with ARF. Paraproteinemias are more prevalent in older populations; therefore, we included patients aged >50 years, and because of elevated FLC levels in patients with diabetes, liver diseases, and collagen tissue diseases, such patients were excluded. 

It has been reported that 24-h urine specimens can be obtained in 50% of patients [16,17]; however, in the present study urine specimens could not be evaluated because the patients presented with oligo and anuria. Studies have reported that cast nephropathy is the predominant cause of renal failure in patients with paraproteinemia that present with ARF [14,15]. In the present study, however, histologic types of ARF in the patients with and without MM could not be determined due to hemorrhagic diathesis and other medical conditions that contraindicated renal biopsy. Based on the published reference range for the FLC κ:λ ratio (0:26-1:65), it had a sensitivity of 71% and specificity of 96%, and a positive and negative predictive value of 62.9% and 97.3%, respectively, for the diagnosis of MM. In the present study there weren’t any significant differences in the sensitivity, specificity, or positive and negative predictive values between the FLC κ:λ ratio and SPE. 

Hutchison et al. reported that based on the published reference range, the sensitivity of the FLC κ:λ ratio was 100% and its specificity was 93% [17]. The specificity of the FLC κ:λ ratio increased to 99% with no change in sensitivity when the renal reference range used was 0:37- 3:1. Moreover, based on the published reference range Katzmann et al. reported that the FLC κ:λ ratio has a sensitivity of 97%, specificity of 100%, and positive and negative predictive values of 100% and 99%, respectively [11]. The authors used nephelometric methods in these reports. Yet, Jaslowski et al. [18] reported that the FLC κ:λ ratio has lower sensitivity than SIFE (91.4% versus 72%) when turbidimetric methods are used, as in the present study. They examined 483 consecutive serum samples with abnormal SIFE findings and reported some of the samples had a normal FLC κ:λ ratio. As such, they suggest that in routine examination the FLC κ:λ ratio has lower diagnostic value. In the present study we also observed low specificity and sensitivity with turbidimetric methods; however, the present study population was small and this method was used firstly in our hospital. 

FLC measurement is accepted as sufficiently sensitive for diagnosing non-secretory myeloma [19]; however, in the present study patients with MM confirmed via bone marrow biopsy findings that had normal SPE and SIFE findings also had a normal FLC κ:λ ratio (1:14). Despite the advantages of FLC assays, they do have some limitations, including reagent lot variability, low ratios due to high antigen levels, innumerable epitopes, and excess polymerization [20,21]. Elevated FLC κ and λ levels correlate strongly with declining kidney function [10]. To address the effect of renal function on FLC levels, patients assessed according to GFR as below and above to eGFR as 15 mL·min–1·1.73 m–2. κ levels had a statistical significance between two groups but λ and κ/λ values had no significance. 

Based on Pearson’s correlation analysis in the present study, creatinine was positively correlated with FLC κ (P = 0.019, r = 0.27) and λ (P = 0.018, r = 0.272) levels, and eGFR was inversely correlated with FLC κ (P = 0.023, r = –0.262) and λ (P = 0.034, r = –0.246) levels. These results are consistent with other reports that emphasize the correlation between FLC levels and renal function [10]. The present study included only 82 patients and FLC levels were assessed using turbidimetric methods. The FLC κ:λ ratio had lower sensitivity and specificity than SPE and SIFE in the patients with MM that presented with ARF. In conclusion, additional, well-designed large-scale prospective studies are required to further delineate the diagnostic utility of the FLC κ:λ ratio. 

**Conflict of Interest Statement**

The authors of this paper have no conflicts of interest, including specific financial interests, relationships, and/ or affiliations relevant to the subject matter or materials included.

## Figures and Tables

**Table 1:**
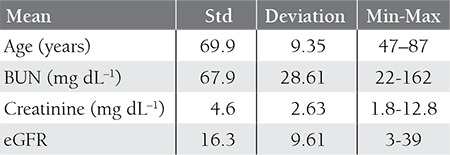
Baseline patient demographic and biochemical data.

**Table 2:**
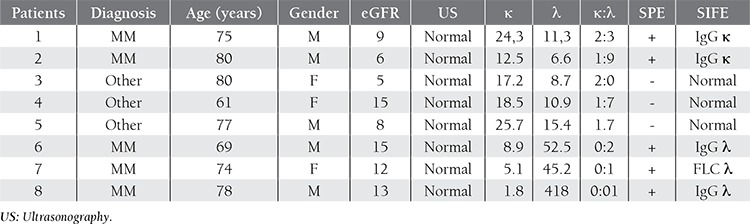
Characteristics of patients with an abnormal FLC k:λ ratio.

**Table 3:**
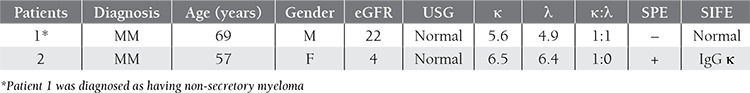
Patients with a normal FLC k:λ ratio diagnosed as MM (false negative cases).

**Table 4:**
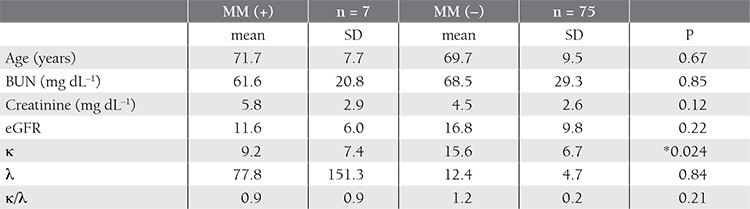
Comparison of the patients with and without MM.

**Table 5:**
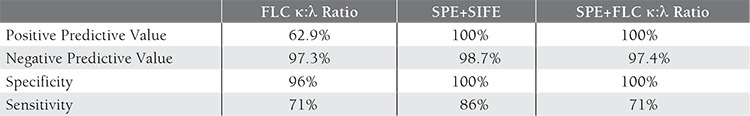
The sensitivity, specificity, and positive and negative predictive values for SPE+SIFE, the FLC k:λ ratio, and the FLC k:λ ratio+SPE.

**Table 6:**
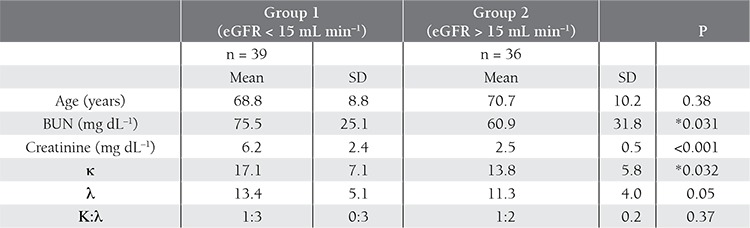
Patients assessed according to eGFR, after exclusion of MM patients.

**Figure:**
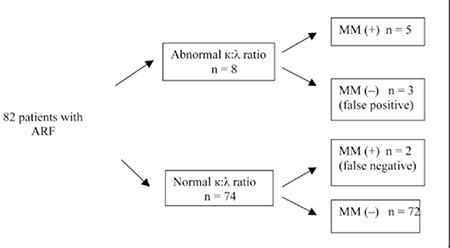
Flow diagram of FLC measurement results and MM diagnoses.
